# Contributions to Obstetric Jurisprudence

**Published:** 1859-11

**Authors:** Horatio R. Storer

**Affiliations:** Boston


ART. I.—*Contributions to Obstetric Jurisprudence.* By HORATIO R. STORER, M.D., of Boston.

### CRIMINAL ABORTION. (CONCLUDED.)

### VIII. CAN IT BE AT ALL CONTROLLED BY LAW?

To this important question I do not hesitate to give an unqualified answer in the affirmative. The fact that criminal abortion is not controlled by law anywhere, cannot be entertained as a valid argument to the contrary of this assertion; for it is equally the fact, as we have seen, that laws against abortion do not as yet exist, which are in all respects just, sufficient, and not to be evaded.

It is evident that in aiming to suppress this crime, the law should provide not merely for its punishment, but indirectly as well as directly, and so far as possible, for its prevention. The punishment of a crime cannot be just, if the laws have not endeavored to prevent that crime by the best means which times and circumstances would allow,* and this is to be accomplished by a twofold process: by rendering on the one hand its detection more probable, and on the other its punishment more certain.

As indirect though important measures for the former of these ends, we have already mentioned laws for registration,† and against conceal-

---

* BECCARIA, Crimes and Punishments, 104.

† "An efficient and practical remedy for the prevention of this crime would be a law requiring the causes of death to be certified by the physician in attendance, or ment of births and secret burials. As a single proof of their possible influence in this respect, out of many that might be adduced, we instance the fact that in Paris the number of premature fœtuses deposited at the Morgue, during the nine years, from 1846 to 1854, inclusive, was found to exceed by more than two-thirds that of the full decade just preceding, from 1836 to 1845.* To render this difference more apparent, we have compiled the following table:—

| Age of fœtuses deposited. | Ten years: 1836–1845. | Nine years: 1846–1854. |
|---|---|---|
| From 2 to 3 months . . . . . . . | 21 | 58 |
| " 3 to 4 " . . . . . . . | 35 | 73 |
| " 4 to 5 " . . . . . . . | 56 | 102 |
| " 5 to 6 " . . . . . . . | 69 | 82 |
| Total . . . . . . . | 181 | 315 |

Part of this advance, it is true, is attributable to the increase in the population of Paris, and in the prevalence of criminal abortion, but in great measure it is clearly owing to the enforcement of a more rigid law against secret burials. The above remarks are strikingly corroborated by the fact that of trials for the crime, and we must not forget that these bear but a small ratio to the whole number of cases preliminarily investigated,† there were in France, during the latter of these periods, fully four times the number occurring from 1836 to 1845.

The establishment of foundling hospitals, by the State governments, has been urged as a preventive of the crime, and, on the other hand, fears have been expressed lest the same means should increase it. For ourselves, however, and from some experience in such cases, we believe that these fears are groundless, and that with equal justice might they be entertained of every large charity having for its end the improvement, sanitary or otherwise, of the masses of society.

We have quoted a statute existing in Massachusetts, though practically unenforced, against one great agent in the increase of abortion, an abuse

---

where there has been no physician, by one called in for the purpose. In this way the cause of death, both in infants and mothers, could be traced to attempts to procure abortion. In three cases which occurred in Boston, in 1855, the death was reported by friends to be owing to natural causes, and in each it was subsequently ascertained that the patient died in consequence of injuries received in procuring abortion. It is probable that such cases are by no means rare; and if the cause of death were known, an immediate investigation might lead to the detection of the guilty party." (Boston Med. and Surg. Journal, Dec., 1857, p. 365.)

* Register of the Morgue.

† From 1846 to 1850, 188 cases of criminal abortion were discovered in Paris, but for want of proof, only 22 of them were sent to trial. (Comptes Rendus Ann. de la Justice Criminelle.)

of its license by the public press. Were such laws to become general, and to be faithfully executed, and were it also made penal to sell any drug, popularly known as emmenagogue, except as advised by physicians, just as the sale of direct poisons is, or should be, controlled by law, the present system of openly advertising by abortionists would undoubtedly be brought to a close.

In no matter is it of more importance than in cases of suspected criminal abortion, that coroners should be intelligent and well educated medical men; and we could wish that this point might have received especial attention from Dr. Semmes, in his late admirable report to the American Medical Association.* In the sudden excitement of an inquest, the guilty are more likely than at a later period to be off their guard, and evidence may often be elicited at this time, which, at the subsequent trial, it would be impossible to obtain. There can be no question of the importance of this point; the coroner should be skilled in all that pertains to obstetric jurisprudence; and if similar knowledge were more generally possessed by other officers of justice, attorney, juror, and judge, a far greater number of convictions, under a proper law, would be secured.

As regards the more direct statutes, we have already considered their important points.

"In order to render laws effectually preventive," has wisely been said, "they should be consistently framed, and based on justice."† In accordance with this truly axiomatic doctrine, and with various rulings of the courts, already quoted, no proof should be demanded which is not necessitated by the actual character of the crime. We have seen that neither in intent nor in fact is this an attempt against the person or life of the mother. If she die in consequence, the offender is already amenable for it as homicide; in the absence of any special statute, at common law. The crime, both in intent and in fact, is against the life of the child.

The attempt being proved, it is unnecessary that it should have been consummated, not merely the completion of a crime bringing its punishment, but also certain overt acts with intent to the perpetration; nor is it requisite that any injury, specific or general, should have been inflicted upon the person of the mother.

The offence being of equal guilt throughout pregnancy, proof of quickening, the incident, not the inception of vitality,—indicating neither the commencement of a new stage of existence, nor an advance from one stage to another,—‡ and, therefore an element without the slightest intrinsic value, should not be required.

---

* Report on the Medico-legal duties of Coroner. 1857.

† RADFORD, British Record of Obstetric Medicine, vol. i. p. 55.

‡ WHARTON, Criminal Law, 540.

The crime of abortion should be considered to include, as it does in the absolute fact of moral guilt, all cases of attempted or intentionally effected destruction and miscarriage of the product of impregnation; and this, whether it be living or dead, normal or abnormal, which last expression equally comprehends instances of moles, hydatids, extra-uterine conception, acephalous, anencephalous, and other monsters.

Proof should not, as now, be required of intent to destroy the child.* This should be considered shown by the intent to produce miscarriage, in the absence of lawful justification therefor; the act in all stages of pregnancy being attended with great danger to the child, and, in much more than a moiety of the period, necessarily fatal to it.

The attempt being considered criminal, it follows that proof of pregnancy is not necessary, and that conviction should be had though it were proved that pregnancy did not exist,† even that the woman on whom the abortion was attempted, however unlikely, was still a virgin.‡

No discrimination should be made as to the means criminally employed, and an escape thus afforded to the guilty; as we have seen still obtaining in Great Britain and many of our own States.

The mother, almost always "an accessory before the fact," or the principal, should not, as now, be allowed almost perfect impunity. There is no valid reason for such exemption, there is every reason against it. The woman is covered by the laws of most continental nations of Europe,—France, Austria, Germany, Bavaria, and Italy,—and by many of them her punishment, if married, is greatly increased. Similar severity is also exercised in these countries against the father of the fœtus, if he too is implicated in the crime.

To allow that abortion is extenuated in the unmarried, it has been said, will "to the moral and political philosopher appear to have exalted the sense of shame into the principle of virtue, and to have mistaken the great end of penal law, which is not vengeance, but the prevention of crime. Law, which is the guardian and bulwark of the public weal, must maintain a steady and even rigid watch over the general tendencies of human actions."§ But, on the other hand, "the measure of punishment should be proportionate, as nearly as possible, to the temptation to offend, and to the kind and degree of evil produced by the offence."||

We have seen the increase in moral guilt, and of opportunity for commission and for escape, in the case of nurses, midwives, and other classes

---

* Smith *vs.* The State, 33 MAINE, (3 RED.) 48.

† Rex *vs.* Phillips; Regina *vs.* Goodall; Reg. *vs.* Haynes, etc.

‡ TAYLOR, Med. Jurisprudence, p. 386.

§ PERCIVAL, Medical Ethics, p. 84.

|| Ibid., p. 85.

of persons, who, from their profession, are brought more directly into contact with pregnant women. By the penal code of Napoleon the First, remarkable in so many respects for the wisdom of its provisions, an increase of punishment was enacted for abortion criminally induced or advised by physicians, surgeons, or other officers of health, including midwives, or by druggists;* their guilt being enhanced by their greater opportunities and knowledge.

Punishments for the crime of abortion should not, as is now generally the case, be so framed as to render the statute, in fact, if not in name, simply nugatory. Were the murder of adults to be made answerable by merely a year or two in prison, far more convictions than at present would undoubtedly be secured; but it is certain that the instances of the crime would be fearfully increased. We have reason to believe that it is precisely thus with the case in hand.

A standard of justification for the instances of necessary abortion should be fixed by law. If perfection in this respect be impossible, let the nearest approach be made to it that can. Since my remarks upon the relative rights of the mother and fœtus to the chance of life in doubtful cases were published in a former paper of the present series, I have received from Dr. Rattenmann, late of Tübingen, an essay, written by himself, in which this question is discussed at length, and the repetition of abortion upon the same individual, in the early months of pregnancy, is defended. I have carefully considered the several arguments advanced by the gentleman, and am compelled to adhere to the views I have already expressed.

In presenting a report upon the matter, in 1857, by direction of the Suffolk District Medical Society of Massachusetts, the writer offered the draft of a law, prepared after much thought and consultation with legal as well as with medical men, and embodying the suggestions made above. This was intended for the consideration of the Legislature of the State, in the hope that it might be of aid toward a modification of the present defective law.

Having seen no reason to change the opinions then avowed, but, on the contrary, receiving constant confirmation of their truth, I now present the essential portions of that draft, acknowledging most willingly that its wording may, perhaps, with safety, be simplified and condensed; but contending, in all sincerity and earnestness of purpose, that its general tenor is what justice and humanity alike, and imperatively, demand at the hands of society.

"Whoever, with intent to cause and procure the miscarriage of a wo-

* Loc. cit., article 317.

man, shall sell, give, or administer to her, prescribe for her, or advise, or direct, or cause, or procure her to take any medicine, or drug, or substance whatever, or shall use, or employ, or advise any instrument, or other means whatever, with the like intent, unless the same shall have been necessary to preserve the life of such woman, or of her unborn child, and shall have been so pronounced (in consultation) by two competent physicians; and any person, with the like intent, knowingly aiding and assisting such offender or offenders, shall be deemed guilty of felony," etc. etc.; "and if such offence shall have been committed by a physician, or surgeon, or person claiming to be such, or by a midwife, nurse, or druggist, such punishment may be increased at the discretion of the court."

"Every woman who shall solicit, purchase, or obtain of any person, or in any other way procure, or receive, any medicine, drug, or substance whatever, and shall take the same, or shall submit to any operation or other means whatever, or shall commit any operation or violence upon herself, with intent thereby to procure a miscarriage, unless the same shall have been by two competent physicians (in consultation) pronounced necessary to preserve her own life, or that of her unborn child, shall be deemed guilty," etc. etc.; "and if said offender be a married woman, the punishment may be increased at the discretion of the court."

It was also advised that the encouragement of criminal abortion, by publication, lecture, or otherwise, or by the advertisement, sale or circulation of such publication, should be made penal, and that the present well worded statute against the personal advertisements of abortionists, and their nostrums, should be rigorously enforced.

To the words now quoted were added, and they are still applicable, the following:—

"We have aimed at a statute, which, while it better defined this atrocious crime, and covered the usual grounds of escape from conviction, established also the proper standard of competence in all medical questions involving issues of life and death. We believe that it would be the means of preventing much of the present awful waste of human life. But enforce such a law, and the profession would never allow its then high place in the community to be unworthily degraded; nor, as now, would those be permitted, unchallenged, to remain in fellowship, who were generally believed guilty, or suspected even of this crime."*

In the same belief, strengthened by nearly three years careful reflection, that criminal abortion can, to a great extent, be controlled by law, if but the community so will, we proceed to the last division of our subject, and discuss the duty of the medical profession toward the ultimate suppression of the crime.

* Report to Suffolk District Med. Society, May, 1857, p. 12.

### IX. THE DUTY OF THE PROFESSION.

I have stated that the prevalence of criminal abortion is in great measure owing to a seeming neglect of fœtal life on the part of medical practitioners, and that in other degree it is attributable to ignorance by the community of the actual character of the offence, an ignorance of physiological facts and laws; and on both these points abundant proof has been afforded of the truth of my assertions.

I have also stated that medical men, in all obstetric matters, are the physical guardians of women and their offspring; a proposition that none can deny.

We have seen that unjustifiable abortion, alike as concerns the infant and society, is a crime second to none; that it abounds, and is frightfully on the increase; and that on medical grounds alone, mistaken and exploded, a misconception of the time at which man becomes a living being, the law fails to afford to infants and to society that protection which they have an absolute right to receive at its hands, and for the absence of which every individual who has, or can exert, any influence in the matter, is rendered so far responsible.

Under these circumstances, therefore, it becomes the medical profession to look to it, lest the *whole* guilt of this crime rest upon themselves.

And, in the first place, it might be asserted with some truth, that such is indeed the case. For, on the one hand, it was from physicians, as is proved by early medical literature, that the mistaken notions, both of the law and of the people, regarding intra-uterine vitality, were derived; and on the other, the apathy and silence still existing on this subject among medical men, though thousands and hundreds of thousands of human lives are thus directly at stake, and are annually sacrificed, can only be explained on one of two suppositions,—either that we do not yet really believe in the existence of fœtal life, though professing to do so, or that we are too timid or slothful to affirm and defend it. By the one alternative a gross lie seems proved; by the other a degrading and strange inconsistency.

But, I believe, this apparent negligence proceeds only from ignorance of the real duty of the profession. It is my aim, while setting forth a deliberate and carefully prepared opinion upon this point, to inspire, if possible, in my fellow-practitioners throughout the land, somewhat of the holy enthusiasm sure in a good cause to succeed despite every obstacle, and an earnest, uncompromising hostility to this result of combined error and injustice, the permitted increase of criminal abortion.

Enough has already been said to show that there is need of increased vigilance on the part of medical men, lest they themselves become innocent and unintentional abettors of the crime.* If, on the other hand, the community were made to understand and to feel that marriage, where the parties shrink from its highest responsibilities, is nothing less than legalized prostitution, many would shrink from their present public confession of cowardly, selfish and sinful lust. If they were taught by the speech and daily practice of their medical attendants, that a value attaches to the unborn child, hardly increased by the accident of its birth, they also would be persuaded or compelled to a similar belief in its sanctity, and to a commensurate respect.

But it is asked, is our silence wrong? Is there not danger otherwise of increasing the crime? These are the questions not of wisdom, or prudence, or philanthropy, but of an arrant pusillanimity. Vice and crime, if kept concealed, but grow apace. They should be stripped of such protection, and their apologists, thereby their accomplices, condemned. Answers, however, are ready at hand to the questions proposed.

"It is one of the great desiderata of registration, that more particularity should be observed in collecting facts relating to the subject of the still-born."†

"Has it been sought," society demands of the profession, "to account for the peculiarities relating to the still-born, and to combat the causes which in certain circumstances swell their number in so deplorable a manner?"‡

"An honest and fearless expression of these causes and circumstances, on the part of medical men, would bring to light an amount of knowledge that might be useful in checking this horrible and increasing waste of life."§

"Such an exercise of his knowledge, experience, and true moral courage, is not only the province, but the conscientious duty of the physician."||

"It should be constantly borne in mind, that there is here a high and

---

* In this connection, I cannot too strongly deprecate a practice that has lately been proposed, the detection, namely, of the early existence of pregnancy by the administration of ergot. (Boston Med. and Surg. Journal, April, 1859, p. 197.) The use of ergot for this purpose, in however small a dose, would seem utterly unjustifiable.

† Fifteenth Massachusetts Registration Report, 1857, p. 199.

‡ QUETELET, Theory of Probabilities, p. 234.

§ New York Med. Gazette, Editorial; London Medical Times and Gazette, 1850, p. 487.

|| Boston Med. and Surg. Journal, Editorial, 1855, p. 411.

stern morality, that should stimulate the medical profession in the exercise of their utmost effort and ingenuity, with the view to master and disclose the secrets of these villainous practices; the more villainous, because they are very generally conceived in fraud, practiced in deception, upon innocent and unsuspecting victims, and result the most commonly in the destruction of two lives at once."*

"For ourselves, we have no fear that the truth in reference to the crime of procuring abortion, would do aught but good. It would appear that sheer ignorance in many honest people, is the spring of the horrible intrauterine murder which exists among us; why not then enlighten this ignorance? It would be far more effectually done by some bold and manly appeal, than by the scattered influence of honorable practitioners alone. Will not the mischief, by-and-by, be all the more deadly, for delaying exposure and attempting relief?

"Whatever estimate may attach to our opinion, we believe that not only ought these things not so to be, but that the public should know it from good authority."†

"This is a topic which ought to be regarded of the highest interest to the profession and the public."‡

"It is by far the most important subject before the profession, and in its medical as well as moral bearings, appeals alike to our patriotism and humanity."§

"We think the public have very erroneous ideas of the turpitude of this crime, and we deem it our duty, as conservators both of the public health and morals, to set it in a correct light before them."||

"The question of criminal abortion is doubtless one of extreme difficulty; it is not, however, beyond the reach of the enlightened prudence and the firm will of the authorities. It is a subject of such vital import to society in general, that we feel convinced it cannot but awaken the anxious thought of the persons who, from their position, are entrusted with the application of the laws and the control of public morals."¶

* DEAN, Medical Jurisprudence, p. 139.

† Boston Med. and Surg. Journal, Editorial, Dec. 13, 1855.

‡ American Medical Gazette, Editorial, July, 1857, p. 390.

§ Ibid., April 1859, p. 289.

|| Maine Med. and Surg. Reporter, Editorial, June, 1858, p. 39.

¶ DEVILLE, Researches on the proportion of still-born children compared with the mortality of the City of Paris during the thirteen years, 1846–58. Memoirs of the French Academy, 1859.

Whoever shall succeed in fixing upon it the attention it deserves, "has taken a stand in this matter alike creditable to his head and his heart, and we feel that he will receive the hearty thanks of every true physician."*

"The increasing prevalence of infanticide," which is but rare compared with criminal abortion, "its dangerous moral influence, the apathy with which so many regard its spread, and the very considerable difficulty in obtaining conviction, call loudly for reform. The question is one of national importance."†

"With this view of the case before us, I suggest it as our imperative duty to direct the attention of legislators to the importance of enacting a statute in conformity thereto."‡

Such are the confessions, independently given, of high-minded and honorable medical men. No more can be added, no less would have been true.

It must be granted, then, that a bold and manly utterance of the truth, combining as this must, contradiction of the error and denouncement of the crime, should be made by the members of the profession on every occasion. By this course it is plain that a healthier moral tone would be made to prevail in the community, the crime would become of rarer occurrence, and the laws, such as they are, would be more faithfully attempted to be enforced.

But it has been shown that the laws on the subject of criminal abortion are radically imperfect and defective, and that this is attributable, wholly, to a medical cause. We assert, therefore, that not only is it the duty of the profession as individual components of society, but more especially as medical men, to see them amended, and to leave no means untried, no effort unmade, for the attainment of this end.

"Physicians alone," says Hodge in his Introductory Address, "can rectify public opinion, they alone can present the subject in such a manner that legislators can exercise their powers aright in the preparation of suitable laws; that moralists and theologians can be furnished with facts to enforce the truth upon the moral sense of the community, so that not only may the crime of infanticide be abolished, but criminal abortion properly reprehended, and that women in every rank and condition of life

---

* New Hampshire Journal of Medicine, Editorial, July, 1857, p. 216.

† London Lancet, Editorial, July, 1858, p. 66.

‡ TATUM, Virginia Med. Journal, June, 1856, p. 457.

may be made sensible of the value of the fœtus, and of the high responsibility which rests upon its parents."*

It has been stated, indeed publicly avowed by a medical body,† that when a physician "shall become cognizant of any attempt unlawfully to procure abortion, either by persons in the profession or out of it, it shall be his duty immediately to lodge information with some proper legal officer, to the end that such information may lead to the exposure and conviction of the offender."

This doctrine is doubtless true to a great extent, but it cannot be applied to the confidential disclosures of patients themselves, which no man has a right to reveal, unless constrained by the direct command of the court, at which, it has been ruled, even professional secrets must be divulged.‡

It follows, from the evidence we have now adduced, that if it be the duty of the profession to urge upon individuals the truth regarding this crime, it is equally their duty to urge it upon the law, by whose doctrines the people are bound; and upon that people, the community, by whose action the laws are made.

And this should be done by us, if we would succeed in suppressing the crime, not by separate action alone, but conjointly, as the profession, grandly representing its highest claim,—the saving of human life.

Every step toward this end should be hailed with enthusiasm. The late action of the State Society of Massachusetts, directly resulting from professional agitation of the subject, deserves praise and imitation; the body referred to having passed a series of resolutions to the following effect: "That the Fellows of the Massachusetts Medical Society regard with disapprobation and abhorrence all attempts to procure or promote abortion, except in cases where it may be necessary for the preservation of the mother's life; and that no person convicted of such attempt, can, consistently with its by-laws, any longer remain a fellow of the society."§

But the mere passage of resolutions in disapproval of this horrible and so rapidly increasing crime is not sufficient to effect its abatement. Something more is wanted than the testimony of record books, the pointless vote of a board of councillors. There must come a hearty, earnest,

---

* Loc. cit., p. 19.

† The Councillors of the Massachusetts Medical Society. Proceedings of the Society, 1858, p. 77.

‡ PHILLIPS, On Evidence, i. p. 135; RYAN, Medical Jurisprudence, p. 193; STORER, Sen., Introductory Address, 1855, p. 10; SIMPSON, Physicians and Physic, p. 31.

§ Proceedings of the Society, 1858.

and unanimous voice from the mass of the profession; an assertion that criminal abortion, or at least its permitted commission, shall no longer exist.

Too much zeal cannot be shown by physicians in relieving themselves from the weight of responsibility they may have incurred by innocently causing the increased destruction of human life. Let it not be supposed by the public that there is among us, either in theory or practice, any disregard of the unborn child. If such impression have already obtained, from our own negligence, the falsehoods of irregular practitioners, or otherwise, it should at once be removed. Fœtal life ever is, and ever has been, held sacred by all respectable physicians, and whenever criminal abortion has been known to have been advised, perpetrated or abetted by one claiming our honorable name, he has invariably and at once lost all professional standing.

We have seen that it is no trifling matter, this awful waste of human life. It is a subject that demands the best efforts of the whole profession as a body and as men. The crime, no longer practiced in secret, must be met boldly; and met with unanimity, it will be met successfully.

But whether these efforts are to be at once decisive or not, whether they are to be received with the gratitude of the community or its disfavor, is no concern of ours. Our duty is very plain; it is to stand, irrespective of personal consequences, in the breach fast making in the public morality, decency, and conscience, and, to the best of our strength, to defend them.

It might be, it very likely would be, for our immediate pecuniary interest, as a profession, to preserve silence; for we have shown that abortions, of all causes, tend to break down and ruin the health of the community at large. But to harbor this thought, even for a moment, were dishonorable. "I will never set politics against ethics," said Bacon, "for true ethics are but as a handmaid to divinity and religion."

We must take this decided stand, there is no choice; else we are recreant to the high trust we have assumed, and to ourselves. Whether the suppression of abortion be effected or no, one thing is certain, our own hands will have been cleansed of this sea of blood. We shall have declared our abhorrence and our innocence of the crime.

Longer silence and waiting by the profession would be criminal. If these wretched women, these married, lawful mothers, aye, and these Christian husbands, are thus murdering their children by thousands, through ignorance, they must be taught the truth; but if, as there is reason to believe is too often the case, they have been influenced to do so by fashion, extravagance of living, or lust, no language of condemnation can be too strong.

Let us, then, meet the issue earnestly and boldly. Silence and patient expectance have been fairly tried; the disease is not self-limited; the evil, instead of working its own cure, has assumed a gigantic, an awful growth.

Abstract discussions of this matter, by ourselves, and within the closed doors of our several societies, no longer avail. We are all agreed upon the guilt of abortion; we ever have been. Our prayers for its suppression have not been answered, for they have hitherto been offered with inactive hand.

We should, as a profession, openly and with one accord appeal to the community in words of earnest warning; setting forth the deplorable consequences of criminal abortion, the actual and independent existence, from the moment of conception, of fœtal life. And that the effort should not be one of words merely, we should, as a profession, recommend to the legislative bodies of the land, the revision and subsequent enforcement of all laws, statutory or otherwise, pertaining to this crime,—that the present slaughter of the innocents may to some extent, at least, be made to cease. For it is "a thing deserving all hate and detestation, that a man in his very originall, whiles he is framed, whiles he is enlived, should be put to death under the very hands and in the shop of nature."*

In conclusion; a committee, consisting of Drs. Blatchford, of New York, Hodge, of Pennsylvania, Pope, of Missouri, Barton, of South Carolina, Lopez, of Alabama, Semmes, of the District of Columbia, Brisbane, of Wisconsin, and the writer, was appointed by the National Medical Association, at its meeting at Nashville, in 1857, to report upon criminal abortion, with a view to its general suppression. The report of this committee, brief, but in strict accordance with the series of papers now ended, has lately been made to the Association, at its session held at Louisville, in May of the present year. The report was accepted, and the resolutions appended to it† were *unanimously* adopted.

---

* Man Transformed. Oxford, 1653.

† "*Resolved*, That while physicians have long been united in condemning the procuring of abortion, at every period of gestation, except as necessary for preserving the life of either mother or child, it has become the duty of this Association, in view of the prevalence and increasing frequency of the crime, publicly to enter an earnest and solemn protest against such unwarrantable destruction of human life.

"*Resolved*, That in pursuance of the grand and noble calling we profess,—the saving of human life,—and of the sacred responsibilities thereby devolving upon us, the Association present this subject to the attention of the several legislative assemblies of the Union, with the prayer that the laws by which the crime of abor-

In behalf of the committee, of whom he had the honor to be chairman, the writer cannot close this portion of his labors without thanking the physicians of the land, represented as they are by the Association, for their hearty and noble response to the appeal that has been made them. He would express, were it possible, the gratitude not of individuals, but society; for by this act the profession has again been true to "its mighty and responsible office of shutting the great gates of human death."

tion is attempted to be controlled may be revised, and that such other action may be taken in the premises as they in their wisdom may deem necessary.

"*Resolved,* That the Association request the zealous co-operation of the various State medical societies in pressing this subject upon the legislatures of their respective States, and that the president and secretaries of the Association are hereby authorized to carry out, by memorial, these resolutions."—*Transactions of the Am. Med. Association*, 1859, vol. xii. p. 75.

